# Comparative analgesic efficacy of superficial parasternal intercostal plane block versus erector spinae plane block after cardiac surgery in Türkiye: protocol for a multicentre randomised non-inferiority trial

**DOI:** 10.1136/bmjopen-2026-127382

**Published:** 2026-11-09

**Authors:** Burhan Dost, Cengiz Kaya, Zeliha Aslı Demir, Mukadder Orhan Sungur, Alparslan Turan

**Affiliations:** 1 Department of Anesthesiology and Reanimation, Ondokuz Mayis University Faculty of Medicine, Samsun, Türkiye; 2 Department of Anesthesiology and Reanimation, Ankara Bilkent City Hospital, University of Health Sciences, Ankara, Türkiye; 3 Department of Anesthesiology and Reanimation, İstanbul University, İstanbul Faculty of Medicine, İstanbul, Türkiye; 4 Center of Outcomes Research, The University of Texas Health Science Center at Houston, Houston, Texas, USA; 5 Outcomes Research Consortium, Houston, Texas, USA

**Keywords:** Pain management, Cardiac surgery, Adult anaesthesia

## Abstract

**Introduction:**

Median sternotomy is associated with substantial acute postoperative pain, which may impair respiratory function, delay mobilisation and increase opioid exposure. The superficial parasternal intercostal plane (SPIP) block directly targets the anterior cutaneous branches of the intercostal nerves supplying the sternotomy region, whereas the erector spinae plane (ESP) block is performed posteriorly and relies on extensive interfascial spread. Direct, adequately powered comparisons of these approaches after cardiac surgery remain limited. This trial will test whether bilateral SPIP block is non-inferior to bilateral ESP block with respect to cumulative intravenous morphine milligram equivalent (IV-MME) consumption during the first 24 hours after extubation.

**Methods and analysis:**

This multicentre, assessor-blinded, parallel-group randomised non-inferiority trial will enrol 308 adults aged 18–80 years undergoing first-time on-pump cardiac surgery through median sternotomy. Participants will be allocated 1:1, stratified by centre, to a bilateral single-shot SPIP block or a bilateral single-shot ESP block before induction of general anaesthesia. Both groups will receive a standardised perioperative anaesthetic and postoperative multimodal analgesic regimen, including intravenous patient-controlled morphine analgesia after extubation. The primary outcome is cumulative opioid consumption from extubation to 24 hours after extubation, expressed as IV-MME. The three prespecified secondary outcomes are time-weighted average pain intensity during coughing over 0–24 hours after extubation, cumulative 0–12-hour postextubation IV-MME consumption and Quality of Recovery-15 (QoR-15) scores at 24 hours after extubation. Other exploratory outcomes include resting pain, QoR-15 at hospital discharge and chronic postsurgical pain at 3 months.

The primary non-inferiority analysis will be performed in both the intention-to-treat and per-protocol populations using the between-group difference in mean cumulative 24-hour IV-MME consumption, expressed as SPIP minus ESP, with a two-sided 95% CI. The primary opioid-consumption comparison will be conditional on survival through the complete assessment window. Assuming a true between-group difference of zero, a non-inferiority margin of 3 mg IV-MME, a common SD of 6.48 mg, 95% power and a one-sided α of 0.025, 123 evaluable participants per group will be required. Allowing for 20% attrition, 308 participants (154 per group) will be enrolled.

**Ethics and dissemination:**

Central ethics approval was obtained from the Ondokuz Mayıs University Clinical Research Ethics Committee (2026/392; 18 March 2026). Approval will also be obtained at each participating centre before recruitment. Written informed consent will be obtained from all participants. Results will be submitted to a peer-reviewed journal and presented at national and international scientific meetings regardless of the direction or magnitude of the findings.

**Trial registration number:**

NCT07780773.

**STRENGTHS AND LIMITATIONS OF THIS STUDY**

- The multicentre randomised design and centre-stratified concealed allocation will improve generalisability and reduce selection bias.
- Both interventions will be delivered by clinicians with substantial prior experience, using prespecified techniques and a common perioperative analgesic pathway.
- The trial includes patient-centred short-term recovery outcomes and chronic postsurgical pain assessments at 3 months.
- Variation in cardiac procedures and unavoidable centre-level differences may introduce heterogeneity, which will be addressed through stratified randomisation, standardisation, mixed-effects analysis and prespecified sensitivity analyses.

## Introduction

 Acute postoperative pain remains a major clinical challenge after cardiac surgery. Pain related to median sternotomy, chest drains and tissue retraction may impair deep breathing and effective coughing, restrict mobilisation and increase opioid requirements, thereby contributing to pulmonary complications and delayed recovery. Contemporary enhanced recovery pathways for cardiac surgery therefore emphasise multimodal, opioid-sparing analgesia, with ultrasound-guided regional analgesic techniques increasingly incorporated into perioperative care.^1–3^

Thoracic fascial plane blocks offer alternatives to neuraxial and paravertebral techniques, which may be less attractive for patients receiving systemic anticoagulation and at risk of haemodynamic instability.^4^ The erector spinae plane (ESP) block is performed by depositing local anaesthetic deep to the erector spinae muscle. Its clinical effect is attributed to craniocaudal interfascial spread and variable extension towards the paravertebral space. The block is technically accessible and has been widely adopted for thoracic and cardiac surgery.^5^

The superficial parasternal intercostal plane (SPIP) block is an anterior chest wall technique in which local anaesthetic is deposited between the pectoralis major and external intercostal muscles. This approach targets the anterior cutaneous branches of the intercostal nerves, which provide substantial sensory innervation to the median sternotomy region.^6^ By acting closer to the principal source of sternotomy-related nociception, SPIP block may provide more anatomically focused analgesia than an ESP block. The clinical question is whether SPIP block can offer an alternative to ESP block without an unacceptable increase in postoperative opioid requirements, while allowing block placement in the supine position without repositioning for a posterior approach.

Despite increasing use of both techniques, direct comparative evidence remains limited. A recent single-centre, randomised controlled trial compared ESP and SPIP blocks with standard analgesic care in patients undergoing cardiac surgery.^7^ Although both blocks were associated with reductions in postoperative pain intensity, cumulative opioid consumption did not differ significantly among groups. Importantly, the trial was not designed to establish the non-inferiority of SPIP compared with ESP, leaving uncertainty regarding their comparative analgesic effectiveness. The Superficial PARasternal intercostal plane block versus Erector spinae plane block (SPARE) trial aims to address this evidence gap through a multicentre, randomised non-inferiority design using standardised perioperative analgesia and cumulative intravenous morphine-equivalent consumption during the first 24 hours after extubation as the primary outcome. We hypothesise that SPIP block will be non-inferior to ESP block with respect to mean cumulative intravenous morphine milligram equivalent (IV-MME) consumption during the 24 hours following extubation, using a prespecified margin of 3 mg IV-MME.

## Methods

### Study design

This is a multicentre, parallel-group, assessor-blinded randomised controlled non-inferiority trial with two intervention groups and a 1:1 allocation ratio. The protocol has been prepared in accordance with the Standard Protocol Items: Recommendations for Interventional Trials (SPIRIT) 2025 statement.^8^ Recruitment is planned from December 2026 to September 2027, with overall study completion planned for December 2027. Figure 1 presents the participant flow diagram, and table 1 provides the schedule of enrolment, interventions and assessments.

**Figure 1:**
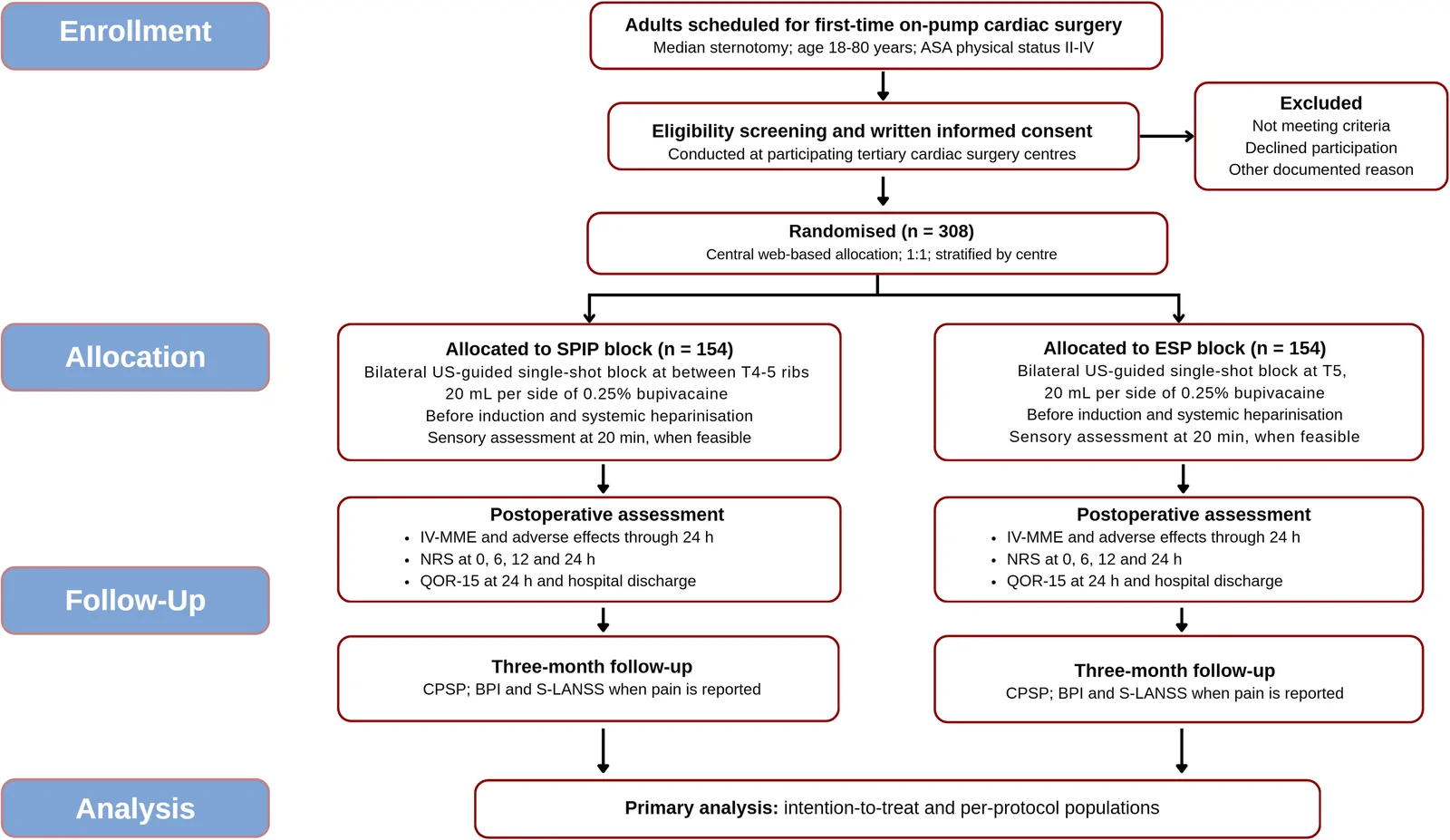
Planned participant flow through the multicentre randomised non-inferiority trial. Eligible adults undergoing first-time on-pump cardiac surgery via median sternotomy will be randomised in a 1:1 ratio to receive either bilateral SPIP block or bilateral ESP block. ASA, American Society of Anesthesiologists; BPI, Brief Pain Inventory; CPSP, chronic postsurgical pain; ESP, erector spinae plane; IV-MME, intravenous morphine milligram equivalents; NRS, Numerical Rating Scale; QoR-15, 15-item Quality of Recovery questionnaire; S-LANSS, Self-Report Leeds Assessment of Neuropathic Symptoms and Signs; SPIP, superficial parasternal intercostal plane; US, ultrasound.

**Table 1:** Schedule of enrolment, interventions and assessments

Study procedure	Screening/preoperative	Randomisation	Block/intraoperative	0-hour	6-hour	12-hour	24-hour	ICU discharge	Hospital discharge	3 months
Eligibility screening	X									
Informed consent	X									
Baseline data	X									
QoR-15	X						X		X	
Randomisation		X								
SPIP or ESP intervention			X							
Sensory block assessment			X							
NRS at rest and during coughing				X	X	X	X			
Opioid consumption						X	X			
Rescue analgesia				X	X	X	X			
PONV and opioid-related adverse effects				X	X	X	X			
Time to extubation				X						
Adverse events			X	X	X	X	X	X	X	X
ICU length of stay								X		
Hospital length of stay									X	
BPI and S-LANSS										X

The 0-hour, 6-hour, 12-hour and 24-hour assessments are referenced to extubation. The 0-hour assessment will be performed as soon as the participant is awake and able to respond. Opioid consumption represents cumulative administration from extubation to 12 and 24 hours, respectively.

BPI, Brief Pain Inventory; ESP, erector spinae plane; ICU, intensive care unit; NRS, Numerical Rating Scale; PONV, postoperative nausea and vomiting; QoR-15, 15-item Quality of Recovery questionnaire; S-LANSS, Self-report Leeds Assessment of Neuropathic Symptoms and Signs; SPIP, superficial parasternal intercostal plane.

### Study setting

The Department of Anesthesiology and Reanimation at Ondokuz Mayıs University will serve as the coordinating centre. Recruitment will take place at tertiary cardiac surgery centres in Türkiye. A current list of participating centres and site investigators will be maintained by the coordinating centre and reported in the final publication. Before activation, each centre will confirm local ethics approval, investigator training, availability of the electronic data capture system and capacity to follow the prespecified perioperative pathway.

### Trial registration

The trial was prospectively registered at ClinicalTrials.gov before enrolment of the first participant (registration number: NCT07780773).

### Eligibility criteria

#### Inclusion criteria

- Age 18–80 years.
- American Society of Anesthesiologists (ASA) physical status II–IV.
- Scheduled first-time on-pump cardiac surgery through a median sternotomy, including coronary artery bypass grafting, valve repair or replacement or another open cardiac procedure requiring sternotomy.
- Ability to understand postoperative pain and recovery assessments and to use an intravenous patient-controlled analgesia (PCA) device.
- Provision of written informed consent.

#### Exclusion criteria

- Emergency surgery.
- Previous median sternotomy.
- Minimally invasive cardiac surgery without median sternotomy.
- Known allergy or hypersensitivity to bupivacaine, morphine, paracetamol, ondansetron or another protocol medication.
- Chronic opioid use, defined as continuous opioid treatment for at least 30 days at a daily oral morphine equivalent (OME) dose of 15 mg or more.
- Alcohol or substance use disorder that, in the investigator’s judgement, may affect adherence or outcome assessment.
- Clinically significant hepatic dysfunction, defined as alanine aminotransferase or aspartate aminotransferase greater than twice the upper limit of normal.
- Clinically significant renal dysfunction, defined as serum creatinine greater than twice the upper limit of normal.
- Coagulation disorder or antithrombotic treatment that precludes safe block performance according to current institutional guidance.
- Infection at a planned needle insertion site.
- Severe psychiatric illness, cognitive impairment or communication difficulty preventing valid postoperative assessment.
- Pregnancy or breastfeeding.
- Body mass index (BMI) greater than 40 kg/m².
- Refusal to participate.

### Recruitment and informed consent

Potentially eligible patients will be identified during the preoperative anaesthesia assessment. A trained investigator will provide verbal and written study information and allow adequate time for questions. Written informed consent will be obtained before any trial-specific procedure. Screening and recruitment logs will be maintained at each centre. No financial inducement will be offered for participation.

### Randomisation and allocation concealment

After eligibility is confirmed and consent obtained, participants will be randomly assigned in a 1:1 ratio to the SPIP or ESP group using a centrally generated computer-based sequence with randomly varying block sizes. Randomisation will be stratified by participating centre. Allocation will be concealed through a secure web-based randomisation system and released only after participant enrolment and immediately before block performance. Personnel involved in screening will not have access to the sequence.

### Blinding

Participants and the anaesthesiologist performing the block will not be blinded because the interventions involve different injection sites and patient positioning. The blocks will be performed by a dedicated anaesthesiologist who will not participate in subsequent intraoperative anaesthetic management, postoperative intensive care unit (ICU) care or outcome assessment. Intraoperative anaesthesia will be managed by a different anaesthesiologist, and postoperative care, including PCA management and rescue analgesia, will be provided by a separate ICU team. Both the intraoperative anaesthesiologist and the ICU team will remain blinded to treatment allocation. Allocation will not be disclosed during routine handover, and procedure-specific documentation will be maintained separately from the routine clinical records accessed by these teams. Information required for safe care, including the total local anaesthetic dose and time of administration, will remain available. Treatment allocation may be disclosed if clinically necessary, and the reason and timing of any unblinding will be recorded. Participants will be asked not to disclose the block location to the care teams or outcome assessors. Postoperative outcome assessors will remain blinded. The statistician will receive coded treatment groups and remain blinded until the primary analysis is finalised.

### Interventions

#### General principles

All blocks will be performed before induction of general anaesthesia and before systemic heparinisation, under standard monitoring and aseptic conditions, by anaesthesiologists who have previously performed each study technique at least 50 times. Bupivacaine 0.25% will be used in both groups. The total bupivacaine dose, including both bilateral study-block injections and chest-tube infiltration, will not exceed the protocol-specified limit of 3 mg/kg ideal body weight. When the maximum permitted dose is lower than the standard planned dose, the injection volume will be reduced proportionally while maintaining the concentration. Any deviation, incomplete injection or additional regional technique will be recorded.

### SPIP group

Participants allocated to SPIP block will receive bilateral ultrasound-guided single-shot injections between the fourth and fifth ribs. With the participant supine, a high-frequency linear transducer will be positioned parasagittally adjacent to the sternal border. A block needle will be advanced in-plane into the fascial plane between pectoralis major and the external intercostal muscle. After hydrodissection confirms the plane and aspiration is negative, 20 mL of 0.25% bupivacaine with epinephrine 1:400 000 will be injected at the fourth intercostal space on each side (planned total volume: 40 mL). In all participants, the surgeon will infiltrate 10 mL of 0.25% bupivacaine around the chest tube. The single-level injection is intended to achieve multisegmental anterior chest-wall sensory coverage through craniocaudal spread within the superficial parasternal plane. Previous sensory-mapping research has reported parasternal block coverage across T2–T6.^9^ However, complete coverage of the sternotomy incision cannot be assumed in every participant. The preinduction cold-sensation assessment will identify a sensory response in the target region but will not establish complete dermatomal coverage.

### ESP group

Participants allocated to the ESP block will receive bilateral ultrasound-guided single-shot injections at the T5 transverse process. With the participant sitting or in the lateral decubitus position, a high-frequency linear transducer will be placed longitudinally in a parasagittal orientation to identify the T5 transverse process and overlying erector spinae muscle. A block needle will be advanced in-plane to the fascial plane deep to the erector spinae muscle. After hydrodissection confirms the plane and aspiration is negative, 20 mL of 0.25% bupivacaine with epinephrine 1:400 000 will be injected on each side (planned total volume: 40 mL). Also, the surgeon will infiltrate 10 mL of 0.25% bupivacaine around the chest tube.

### Assessment of block success and protocol deviations

Cold sensation will be assessed 20 min after block completion and before induction whenever clinically feasible. Responses will be categorised as normal, reduced or absent compared with an unblocked reference region. Assessment will focus on the parasternal anterior chest wall for SPIP block and the anterior and anterolateral thoracic wall for ESP block, as feasible. Responses will be documented separately for each side without formal dermatome-by-dermatome mapping. Technical failure will be defined as inability to complete the assigned bilateral injections in the intended fascial planes. Sensory response will be classified on each side as present when cold sensation is reduced or absent in the assessed target region, and absent when cold sensation remains normal throughout that region. Bilateral absence of a sensory response will constitute sensory block failure; a response on only one side will be recorded as a unilateral sensory response. An assessment that cannot be performed will be recorded as not assessable rather than as block failure. These assessments describe cutaneous sensory responses and do not establish complete analgesic coverage. Participants with technical or sensory failure will remain in their randomised group for the intention-to-treat analysis, subject to the previously specified outcome-availability and estimand provisions. Technical failure will constitute a major protocol deviation and result in exclusion from the per-protocol population. Sensory failure, a unilateral sensory response or an unavailable sensory assessment alone will not result in per-protocol exclusion when the assigned bilateral injections have otherwise been completed according to protocol.

### Safety precautions for regional blocks

All study blocks will be completed before systemic heparinisation. A minimum interval of 30 min between block completion and systemic heparin administration will be targeted whenever clinically feasible. Participants will be observed for immediate complications. Resuscitation equipment and lipid emulsion for the treatment of local anaesthetic systemic toxicity will be immediately available. Suspected pneumothorax, bleeding, neurological deficit or local anaesthetic systemic toxicity will be managed according to institutional protocols and reported as adverse events.

### Standardised perioperative management

Perioperative management will be standardised across centres as far as clinically feasible. Following invasive arterial pressure monitoring, anaesthesia will be induced with intravenous propofol 0.5–2 mg/kg and remifentanil 0.5 µg/kg as a bolus followed by an infusion of approximately 0.3 µg/kg/min. Rocuronium 1 mg/kg will facilitate tracheal intubation, with additional 0.1–0.2 mg/kg doses guided by neuromuscular monitoring. Anaesthesia will be maintained with sevoflurane or desflurane at an age-adjusted minimum alveolar concentration of approximately one in an oxygen–air mixture and remifentanil 0.1–0.3 µg/kg/min, titrated to clinical requirements. Bispectral index monitoring will target values of 40–60. Mechanical ventilation will use a tidal volume of 7–8 mL/kg ideal body weight, an inspiratory-to-expiratory ratio of 1:2 and a respiratory rate adjusted to maintain end-tidal carbon dioxide at 30–35 mm Hg. Volatile anaesthesia will also be maintained during cardiopulmonary bypass in both treatment groups at all participating centres. Before recruitment, each centre will confirm compatibility of its delivery equipment and monitoring with this technique and compliance with local requirements.

Fluid, glucose, vasoactive and cardiopulmonary bypass management will follow institutional practice. Postoperative extubation will occur in the ICU according to local criteria, with a target of extubation within 6 hours when clinically appropriate. Non-protocol analgesic adjuncts likely to influence postoperative pain outcomes—including ketamine, dexmedetomidine, intravenous lidocaine or magnesium infusions and additional regional analgesic procedures—will be avoided unless clinically indicated. Their use and indication will be recorded.

### Postoperative analgesia

All participants will receive intravenous morphine 0.05 mg/kg ideal body weight intraoperatively. Intravenous paracetamol 15 mg/kg will be administered at skin closure and every 6 hours postoperatively, subject to local maximum-dose and organ-function restrictions. After extubation and recovery of adequate consciousness, PCA will be initiated using morphine 1 mg per demand, a 6-minute lockout, no background infusion and a maximum of 20 mg in 4 hours.

If pain persists at a Numerical Rating Scale (NRS) score of ≥4 despite appropriate PCA use, the blinded ICU team will administer 1 mg intravenous morphine as rescue analgesia, provided that the safety criteria below are met. Pain and safety parameters will be reassessed 15 min after each rescue dose and before any subsequent dose. If the NRS score remains ≥4, a further 1 mg dose may be administered after reviewing recent PCA and rescue opioid administration.

Sedation will be assessed using the Richmond Agitation–Sedation Scale (RASS). Rescue morphine will be withheld if systolic blood pressure is <90 mm Hg, clinically significant haemodynamic instability is present, respiratory rate is <10 breaths/min, apnoea or clinical hypoventilation occurs or RASS is ≤−2. These findings will prompt immediate physician assessment and review of ongoing opioid administration, including PCA. Oxygen saturation will be monitored, and values below the prescribed patient-specific target will prompt reassessment before further dosing. Persistent pain despite repeated rescue doses will require physician review rather than automatic continuation of boluses. Pain scores, blood pressure, respiratory rate, oxygen saturation, RASS scores, rescue doses, administration times and reasons for withholding treatment will be recorded. All PCA-delivered morphine and rescue opioids administered within the primary assessment window will be recorded and converted to IV-MME for inclusion in the primary outcome.^10^

### Postoperative nausea and vomiting and opioid-related adverse effects

Dexamethasone 8 mg intravenously will be administered at induction and ondansetron 0.15 mg/kg (maximum 8 mg) approximately 20 min before skin closure. Postoperative nausea and vomiting (PONV) will be graded from 0 (none) to 4 (more than one episode of vomiting). Ondansetron 4 mg intravenously will be used as rescue treatment for a score of 2 or higher or earlier for distressing nausea. Persistent symptoms will be treated with metoclopramide 10 mg intravenously as a slow infusion, unless contraindicated. Opioid-related adverse effects, including nausea and vomiting, pruritus, sedation and respiratory depression, will also be recorded during the first 24 hours after extubation.

### Outcomes

#### Primary outcome

The primary outcome is cumulative opioid consumption from extubation to 24 hours after extubation, expressed as intravenous MME. This will include all morphine delivered by PCA and all additional opioid rescue analgesia administered during the assessment window. Intraoperative opioids will not form part of the primary outcome but will be reported separately.

*Primary hypothesis:* Bilateral SPIP block will be non-inferior to bilateral ESP block with respect to cumulative IV-MME consumption from extubation to 24 hours after extubation, using a prespecified non-inferiority margin of 3 mg IV-MME.

#### Secondary outcome 1

Time-weighted average (TWA) pain intensity during coughing over the first 24 hours after extubation, calculated from NRS scores obtained at 0, 6, 12 and 24 hours using the trapezoidal method. A between-group difference of 1 point on the NRS scale will be considered clinically meaningful.

#### Secondary outcome 2

Cumulative opioid consumption during the first 12 hours after extubation, expressed as IV-MME, including all PCA-delivered morphine and additional opioids administered during this period.

#### Secondary outcome 3

Quality of recovery, assessed using the Quality of Recovery-15 (QoR-15) questionnaire at 24 hours after extubation.

#### Other outcomes

Other prespecified exploratory outcomes will include TWA pain intensity at rest over the first 24 hours after extubation; incidence of chronic postsurgical pain (CPSP) at 3 months, defined according to International Classification of Diseases, 11th Revision criteria^11^; QoR-15 scores at hospital discharge; rescue analgesic requirements; PONV and other opioid-related adverse effects; time to extubation; ICU and hospital length of stay; technical and sensory block failure; and block-related complications. Prespecified definitions of all study outcomes are provided in online supplemental material 1.

### Outcome definitions and assessment procedures

Pain will be measured on an 11-point NRS, where 0 represents no pain and 10 represents the worst pain imaginable. The 0-hour assessment will be undertaken as soon as the participant is awake and able to respond after extubation. Coughing pain will be assessed during a standardised voluntary cough whenever clinically safe.

CPSP will be defined as pain that develops or increases after surgery, persists for at least 3 months, is localised to the sternotomy area or a relevant referred region, and is not better explained by another cause. Pain severity and interference among participants reporting pain will be assessed using the Brief Pain Inventory (BPI),^12^ and neuropathic pain characteristics will be assessed using the Self-Report Leeds Assessment of Neuropathic Symptoms and Signs (S-LANSS).^13^

### Data collection

Data will be collected prospectively using a standardised electronic case report form developed in an electronic data capture system. The following baseline and perioperative variables will be recorded: age, sex, BMI, ASA physical status, comorbidities, baseline chronic pain status, preoperative QoR-15 score, baseline left ventricular ejection fraction when available, planned and actual cardiac surgical procedure, planned and actual internal mammary artery harvesting, maximal sternal retraction distance, number and location of chest drains, duration of surgery, cardiopulmonary bypass and aortic cross-clamp durations, intraoperative opioid administration and use of analgesic adjuncts, time to extubation and mobilisation, and ICU and hospital discharge times. Postoperative data will include QoR-15 scores, PCA and rescue analgesic consumption, pain intensity, PONV scores, opioid-related adverse effects, block assessment findings, protocol deviations, adverse events and postoperative complications. Opioid-administration records will allow separate calculation of cumulative consumption over 0–12 and 0–24 hours after extubation.

Data will be entered by authorised members of the research team at each participating centre. The database will incorporate predefined data ranges, mandatory fields and automated validation rules to minimise missing or inconsistent entries. Each participant will be assigned a unique study identification number, and no directly identifiable personal information will be included in the analytical dataset. Data completeness and consistency will be reviewed periodically by the coordinating centre, and any discrepancies will be resolved through queries sent to the relevant study site.

### Statistical analysis

#### Non-inferiority design and margin

The primary objective will be to evaluate whether the SPIP block is non-inferior to the ESP block with respect to 24-hour cumulative IV-MME consumption.

### Primary estimand

The target population comprises adults meeting the prerandomisation eligibility criteria and undergoing first-time on-pump cardiac surgery through median sternotomy. The treatment conditions are assignment to bilateral single-shot SPIP block or bilateral single-shot ESP block, each delivered within the standardised perioperative analgesic pathway. The outcome variable is cumulative IV-MME consumption during the 24 hours following extubation, including PCA-delivered morphine and all rescue opioids. The population-level summary measure is the between-group difference in mean cumulative 24-hour IV-MME consumption, expressed as SPIP minus ESP. A treatment-policy strategy will be used for block failure, crossover and escalation of rescue analgesia: available outcomes will be analysed according to randomised allocation, regardless of these events. All rescue opioids administered within the primary assessment window will contribute to the outcome. Delayed extubation will not result in exclusion from the analysis. Participants who require prolonged postoperative mechanical ventilation will remain in their randomised group, and cumulative IV-MME consumption will be measured over the full 24 hours following extubation, irrespective of the time elapsed since surgery. Discontinuation of the assigned intervention will not end outcome follow-up when consent permits. If a participant withdraws permission for further data collection, previously collected data will be retained where permitted and any missing outcome data will be addressed using the prespecified missing-data analyses.

Death before extubation or before completion of the 24-hour postextubation window precludes definition of the full primary outcome. The opioid-consumption contrast will therefore be conditional on survival through this assessment window in each randomised group. Participants who die will remain in the randomised trial population and in mortality and safety reporting, but no complete 24-hour opioid value will be assigned or imputed for them. This survivor-conditional comparison may be affected by postrandomisation selection and will not be interpreted as an unconditional treatment effect in all randomised participants. Deaths before completion of the primary assessment window will be reported separately by treatment group and considered when interpreting the non-inferiority findings.

### The non-inferiority margin

The non-inferiority margin was derived using a fixed-margin approach informed by US Food and Drug Administration guidance and the International Conference on Harmonisation of Technical Requirements for Registration of Pharmaceuticals for Human Use (ICH) E10 guideline, “Choice of Control Group and Related Issues in Clinical Trials”.^14 15^ A previous meta-analysis reported a mean reduction of 35.72 mg in opioid consumption during the first 24 hours after surgery with ESP block compared with control, expressed as OMEs (95% CI 20.57 to 50.88 mg).^16^ Using the prespecified 3:1 oral-to-intravenous morphine conversion ratio,^16^ this corresponded to an estimated benefit of 11.91 mg IV-MME (95% CI 6.86 to 16.96 mg). Preserving 50% of the conservative confidence-limit estimate of 6.86 mg yielded a calculated margin of 3.43 mg, which was rounded down to 3 mg IV-MME. This margin would preserve approximately 56% of that historical estimate, provided that it is applicable to the present population, analgesic regimen and outcome-assessment window. The same meta-analysis reported a sensitivity analysis restricted to four single-shot trials, with a reduction of 31.62 mg OME (95% CI 16.39 to 46.85 mg; I²=95%).^16^ Using the conservative confidence limit from this analysis and the same conversion ratio, the 3-milligram margin would correspond to approximately 45% preservation of the historical effect. This analysis suggests opioid sparing with single-shot ESP in the included trials, but does not establish that a 3-milligram margin preserves at least 50% of the benefit under the present study conditions. The 3-milligram margin is retained on the basis of the overall historical estimate and the investigators’ clinical judgement. Substantial heterogeneity and differences between postoperative and postextubation assessment windows mean that neither calculation establishes the proportion of benefit that would be preserved under the present study conditions.

Clinically, the margin represents the maximum additional mean IV-MME consumption over 24 hours considered acceptable by the investigators in exchange for the practical advantage of supine SPIP placement without repositioning for a posterior block. Under the study regimen, 3 mg corresponds to three delivered 1 mg morphine PCA boluses. This comparison illustrates the magnitude of the margin; it does not establish a validated threshold of clinical unimportance or an acceptable increase for each individual participant. The margin applies to the between-group mean difference. Non-inferiority in opioid consumption will not, by itself, establish equivalent pain relief or recovery, and findings will be interpreted alongside pain scores, QoR-15 scores and opioid-related adverse effects.

### Sample-size calculation

In the same meta-analysis, mean 24-hour opioid consumption in the ESP block group was 27.97±19.43 mg OME.^16^ After conversion to the IV-MME scale using the same 3:1 ratio, the corresponding SD was 6.48 mg.^17^ The non-inferiority margin, sample-size calculation and primary analysis therefore target the between-group difference in mean cumulative 24-hour IV-MME consumption. Assuming a true between-group difference of zero, a non-inferiority margin of 3 mg IV-MME, a common SD of 6.48 mg, 95% power and a one-sided α of 0.025, 123 participants per group will be required for the primary analysis. To account for an anticipated attrition rate of 20%, the target sample size will be increased to 154 participants per group (308 participants in total). Sample size calculations were performed using PASS V.21 (NCSS, Kaysville, Utah, USA). The non-inferiority margin, sample-size calculation and primary analysis are specified on the same scale: the between-group difference in mean cumulative 24-hour IV-MME consumption. Potential skewness will be addressed through a prespecified bootstrap sensitivity analysis targeting the same mean difference.^18^

### Analysis populations

The intention-to-treat population will include all randomised participants and will be the primary analysis population. Participants will be analysed according to randomised group regardless of block failure, crossover or protocol deviation. All randomised participants will be accounted for in the trial analyses and reporting. However, the primary opioid-consumption estimate is conditional on survival through the full assessment window, as specified in the estimand and does not constitute a complete-outcome analysis of every randomised participant. Prolonged postoperative mechanical ventilation, defined as failure to achieve extubation within 8 hours after surgery, will be treated as a prespecified intercurrent event rather than an exclusion criterion. These participants will remain in the intention-to-treat population and will be analysed according to their randomised allocation. For participants who are subsequently extubated, opioid consumption will be recorded over the full 24-hour period following extubation, irrespective of the duration of postoperative ventilation. Delayed extubation alone will not exclude participants from the per-protocol analysis. The safety population will include all participants in whom needle insertion is attempted during a study block and will be analysed by the block received. The per-protocol population will include participants who received the assigned bilateral block without a major prespecified protocol deviation. Major deviations will include failure to receive the assigned bilateral block, receipt of an unassigned regional analgesic technique and other intervention-related deviations that are expected to materially affect the treatment contrast. Missing primary outcome data will not, by itself, be classified as a protocol deviation; participants without an observed primary outcome will be described separately and addressed through the prespecified missing-data analyses. Major protocol deviations will be defined prospectively in the statistical analysis plan before database lock. Absence of a cutaneous sensory response alone will not constitute a major protocol deviation when the assigned bilateral injections have been completed according to protocol.

### Primary outcome analysis

The primary analysis will estimate the difference in mean cumulative 24-hour IV-MME consumption between the SPIP and ESP groups, expressed as SPIP minus ESP, with a two-sided 95% CI. The unadjusted CI will be calculated using the Welch two-sample t method. Non-inferiority will be concluded if the upper confidence limit is below the prespecified margin of 3 mg IV-MME. Analyses will be conducted in both the intention-to-treat and per-protocol populations, and a non-inferiority conclusion will require concordant findings in both populations. To assess robustness to skewness, a supportive analysis will calculate a bias-corrected and accelerated bootstrap 95% CI for the mean difference using 10 000 resamples drawn separately within each treatment group.^19^ The Hodges-Lehmann location-shift estimate and its 95% CI will be reported as an additional supportive analysis; this analysis targets a different summary measure and will not determine the primary non-inferiority conclusion.

### Secondary outcome analyses

TWA pain scores during coughing over 0–24 hours will be analysed separately using an appropriate regression model, with treatment group as the main effect and centre accounted for as a random effect. Between-group differences will be reported with 95% CIs. Repeated NRS measurements at individual time points will additionally be analysed using a mixed-effects model including treatment group, time and the treatment-by-time interaction as a supportive analysis.

Cumulative 0–12-hour postextubation IV-MME consumption will be analysed as a secondary outcome. The between-group mean difference, expressed as SPIP minus ESP, will be reported with a two-sided 95% CI calculated using the Welch two-sample t method. No separate non-inferiority margin will be specified for this outcome, and the analysis will not determine the primary non-inferiority conclusion.

Postoperative QoR-15 scores at 24 hours after extubation and hospital discharge will be analysed using baseline-adjusted mixed-effects regression. The model will include treatment group, postoperative assessment time, the treatment-by-time interaction and preoperative QoR-15 score as fixed effects, with random intercepts for participant and centre. The treatment-by-baseline QoR-15 interaction will be examined only in an exploratory analysis and will not determine the model used for the prespecified 24-hour comparison. The baseline-adjusted between-group contrast at 24 hours after extubation will provide the QoR-15 comparison included in the Bonferroni-adjusted family. The hospital-discharge contrast and treatment-by-time interaction will be interpreted as exploratory analyses. Binary outcomes will be analysed using mixed-effects logistic regression with centre included as a random intercept. Results will be reported as ORs together with model-standardised marginal risks and risk differences where estimable. For rare outcomes or models that fail to converge, exact or penalised regression methods will be used as supportive analyses, as appropriate. Time-to-event outcomes will be summarised using the Kaplan-Meier method and compared using Cox regression, with centre accounted for by stratification or a shared frailty term, when appropriate. Length-of-stay outcomes will be analysed using count or time-to-event methods, depending on the observed distributions. CPSP at 3 months will be evaluated as an exploratory outcome. The proportion of participants reporting CPSP will be presented by randomised group, together with the between-group risk difference and its 95% CI. Deaths and missing 3-month assessments will be reported and will not be classified as absence of pain. BPI and S-LANSS scores among participants reporting pain will be summarised descriptively. A non-significant between-group difference will not be interpreted as evidence of equivalence.

Bonferroni adjustment will be applied to a prespecified family of three secondary comparisons: TWA NRS pain intensity during coughing over 0–24 hours after extubation, cumulative 0–12-hour postextubation IV-MME consumption and QoR-15 scores at 24 hours. The family-wise error rate within this family will be controlled at a two-sided level of 0.05. Bonferroni-adjusted p values will be calculated as min (3×p, 1) and compared with 0.05, equivalent to comparing unadjusted p values with 0.05/3. Effect estimates and nominal 95% CIs will also be reported; these intervals will not be interpreted as multiplicity-adjusted intervals. Other prespecified exploratory outcomes and supportive analyses will be interpreted descriptively.

Non-inferiority testing will remain restricted to the primary 24-hour opioid-consumption outcome. No non-inferiority margins have been specified for NRS or QoR-15. Evidence of worse pain or recovery will qualify the clinical interpretation of opioid non-inferiority, irrespective of whether the corresponding comparison meets the multiplicity-adjusted threshold. Non-significant secondary comparisons will not establish equivalence.

### Covariate adjustment and sensitivity analyses

An adjusted supportive analysis of the primary outcome will use multivariable linear regression, with cumulative 24-hour IV-MME consumption as the dependent variable. The model will include treatment group and participating centre as categorical fixed effects, age and BMI as continuous variables, and sex, planned surgical procedure (isolated coronary artery bypass grafting vs other eligible procedures), planned internal mammary artery harvesting and baseline chronic pain as categorical covariates. The treatment coefficient will represent the adjusted mean difference between SPIP and ESP. A two-sided 95% CI will be calculated using heteroscedasticity-consistent HC3 SEs. Covariates will be retained irrespective of statistical significance, without data-driven variable selection. The same model specification will be applied to the intention-to-treat and per-protocol analysis populations, subject to the previously specified estimand and missing-data provisions. Postrandomisation procedural variables, including surgery duration, cardiopulmonary bypass duration and chest-drain characteristics, will not be included in the prespecified adjusted sensitivity model but may be examined in separate exploratory analyses.

An exploratory sensitivity analysis will be restricted to participants with a documented bilateral cutaneous sensory response. Because this restriction conditions on a postrandomisation response, the findings will be interpreted cautiously and will not determine the primary non-inferiority conclusion. Other sensitivity analyses will examine exclusion of participants receiving non-protocol analgesic adjuncts and robustness to skewness using the prespecified bootstrap analysis. Subgroup analyses, if performed, will be limited and interpreted as exploratory. Prespecified candidate subgroups will include age, sex, BMI, isolated coronary artery bypass grafting versus other surgery and internal mammary artery harvesting versus no harvesting. Treatment-effect heterogeneity will be assessed using interaction terms rather than separate significance testing within individual subgroups.

### Missing data and outcomes truncated by death

Every effort will be made to obtain complete opioid-administration records from PCA devices and medication charts. Missingness, its reasons and the number of participants contributing to each analysis will be reported by randomised group. Delayed extubation alone will not constitute missing outcome data. Participants will be followed until extubation and completion of the subsequent 24-hour assessment window, irrespective of the duration of postoperative ventilation. Inability to use PCA will not itself render the outcome missing: all opioids administered by clinical staff during the assessment window will also be included. Reintubation within this window will not restart or terminate the assessment period. Death before extubation or before completion of the assessment window will be distinguished from missing data among survivors. Opioid doses recorded before death will be retained descriptively but will not be treated as a complete 24-hour outcome, extrapolated to 24 hours or replaced with zero. These deaths will not be included in the multiple-imputation procedure. Among participants who survive the full assessment window, the primary available-case analysis will use complete observed outcomes according to randomised allocation. If any primary outcome values are missing, multiple imputation by chained equations will be performed as a sensitivity analysis under a missing-at-random assumption, using at least 50 imputed datasets. The imputation model will include treatment group, centre, prespecified baseline prognostic variables and available opioid-consumption and clinical measurements predictive of the missing outcome or missingness. Predictive mean matching will be used for cumulative opioid consumption to avoid imputing negative values. Mean differences and their variances will be combined using Rubin’s rules. Departures from the missing-at-random assumption will be examined using a delta-adjusted tipping-point analysis. Imputed consumption in the SPIP group will be progressively increased relative to the ESP group, and the shift at which the upper 95% confidence limit reaches or exceeds the 3-milligram non-inferiority margin will be reported.

### Data management and quality assurance

Study data will be entered into a central electronic data capture system using standardised electronic case report forms. Participants will be identified only by a unique study code. Range checks, logical consistency checks and mandatory fields will be built into the database. The coordinating centre will review data completeness and plausibility, issue queries and monitor protocol adherence. Source data verification may be performed on a risk-based sample. Database access will be role-based and password-protected. The final dataset will be locked only after all critical queries have been resolved and the statistical analysis plan signed.

### Confidentiality, data retention and access

The reidentification key will remain securely stored at each participating centre and will not be transferred to the coordinating analysis dataset. Trial records will be retained for the period required by applicable Turkish regulations and institutional policy. During trial conduct, access to the study database will be restricted to authorised members of the trial team. After publication, external access to de-identified data will be considered in accordance with the time frame and access criteria specified in the Data availability statement.

### Monitoring and safety oversight

At each centre, the principal investigator will identify, document and report adverse events and serious adverse events. An independent Data and Safety Monitoring Committee, or an independent safety monitor if a full committee is not feasible, will periodically review accumulating safety information. The oversight body will be independent of the investigators responsible for recruitment and block delivery. Its membership, meeting frequency, access to unblinded data and recommendation process will be described in a separate charter before recruitment.

Events of special interest include pneumothorax, vascular injury, major bleeding or haematoma, infection, local anaesthetic systemic toxicity and persistent neurological deficit. Serious adverse events will be reported according to institutional and regulatory requirements and promptly communicated to the coordinating centre and safety oversight body. Because both interventions are established regional techniques and the trial is not designed for sequential efficacy monitoring, no formal interim efficacy stopping rule is planned. Safety-related suspension or termination may be recommended if an unexpected pattern of serious block-related harm emerges.

### Withdrawal and discontinuation

Participants may withdraw consent at any time without affecting clinical care. Investigators may discontinue trial procedures when required for safety, urgent surgery, a change in operative approach or another clinical reason. Unless the participant withdraws permission for further use, data collected before withdrawal will remain in the analysis. Participants who discontinue the assigned intervention will continue outcome follow-up whenever consent permits. Reasons for withdrawal and discontinuation will be recorded.

### Patient and public involvement

Patients and members of the public were not involved in formulating the research question, selecting outcomes or designing the protocol. The outcomes were chosen to reflect clinically important domains, including pain, opioid exposure, quality of recovery and persistent pain. Participants will not be involved in recruitment or trial conduct. A plain-language summary of the findings will be made available to participants on request after publication.

### Ethics and dissemination

Central ethics approval was granted by the Ondokuz Mayıs University Clinical Research Ethics Committee (decision 2026/392; 18 March 2026). Each participating centre will obtain local approval before recruitment. The study will be conducted in accordance with the Declaration of Helsinki, Good Clinical Practice principles and applicable data protection requirements. Written informed consent will be obtained from each participant by a trained study investigator before any trial-specific procedure. Potential participants will receive verbal and written information about the trial and will be given adequate time to ask questions and consider participation before providing consent.

The findings will be submitted to a peer-reviewed journal and presented at national and international scientific meetings regardless of the direction or magnitude of the results. Authorship will follow the International Committee of Medical Journal Editors criteria. No professional writer will be granted authorship. The final report will follow Consolidated Standards of Reporting Trials (CONSORT) 2025 and relevant extensions.^18^ Protocol deviations and amendments will be transparently reported.

## Discussion

This multicentre randomised controlled non-inferiority trial will directly compare two commonly used thoracic fascial plane blocks for postcardiac surgery analgesia following median sternotomy. Although both the SPIP block and the ESP block have been increasingly incorporated into multimodal analgesic pathways,^20^ robust head-to-head evidence remains limited. This trial will estimate whether mean 24-hour postextubation IV-MME consumption with SPIP is within the prespecified non-inferiority margin relative to ESP. A finding of non-inferiority would support consideration of SPIP as an alternative within the studied pathway, provided that pain, recovery and safety findings are also clinically acceptable. The 3-month assessment will provide exploratory information on CPSP and inform future studies. As the trial is not specifically powered for this outcome, between-group estimates will be interpreted cautiously, with emphasis on their precision. The multicentre design, standardised perioperative analgesic pathway, blinded outcome assessment and inclusion of both acute and longer-term patient-reported outcomes represent important strengths. The 24-hour primary endpoint evaluates overall opioid requirements during the first day after extubation and does not imply uninterrupted sensory blockade. The 0–12-hour MME outcome will provide complementary information on earlier consumption; however, delayed extubation may also shift this shorter window beyond the period of greatest block effect. Interpretation depends on the applicability of historical ESP-versus-control evidence to the present regimen and postextubation assessment window. If the ESP effect is smaller under these conditions, the 3-milligram margin may preserve less benefit or permit its complete loss. Without a no-block or sham control, the trial cannot establish either block’s added benefit over multimodal analgesia. Non-inferiority will therefore apply only to opioid consumption under the study conditions, not to overall analgesic equivalence. Dexamethasone, administered in both groups for PONV prophylaxis, may also contribute to analgesia and attenuate between-group differences in opioid consumption, potentially favouring a finding of non-inferiority. An anticipated limitation is that participants and block-performing anaesthesiologists cannot be blinded because of differences in patient positioning and block technique. To reduce performance bias, the block-performing anaesthesiologist will not participate in subsequent care, and the clinicians responsible for intraoperative anaesthesia and postoperative ICU analgesia will remain blinded to treatment allocation; however, inadvertent disclosure by participants remains possible. Variability in surgical procedures, institutional practices and postoperative recovery pathways may also introduce heterogeneity, although centre-stratified randomisation and adjusted analyses are planned to minimise this effect.

The findings of SPARE trial may help define the most appropriate fascial plane block for patients undergoing cardiac surgery through median sternotomy and may contribute to the development of more standardised, opioid-sparing perioperative analgesic pathways.

## Supplementary material

10.1136/bmjopen-2026-127382 online supplemental table 1
