# Supplementary material for: Comparative analgesic efficacy of superficial parasternal intercostal plane block versus erector spinae plane block after cardiac surgery in Türkiye: protocol for a multicentre randomised non-inferiority trial
Source: BMJ Open. 2026 Nov 9;16(11):e127382. doi: 10.1136/bmjopen-2026-127382 (PMC13756325; doi:10.1136/bmjopen-2026-127382)
Supplement: Supplementary file 1 [file bmjopen-2026-127382.reviewer_comments.pdf]

PEER REVIEW HISTORY

BMJ Open publishes all reviews undertaken for accepted manuscripts. Reviewers are asked to complete a checklist review form and are provided with free text boxes to elaborate on their assessment. These free text comments are reproduced below.

ARTICLE DETAILS

Title (Provisional)

Comparative analgesic efficacy of superficial parasternal intercostal plane block versus erector spinae plane block after cardiac surgery in Türkiye: protocol for a multicentre randomised non-inferiority trial

Authors

Dost, Burhan; Kaya, Cengiz; Demir, Z. Asli; Orhan Sungur, Mukadder; Turan, Alparslan

---

VERSION 1 - REVIEW

Reviewer 1

Name Lei, Chong

Affiliation Fourth Military Medical University, Department of Anesthesiology and Perioprative Medicine, Xijing Hospital

Date 13-Sep-2026

COI NA

This is a multicentre, parallel-group, assessor-blinded randomised non-inferiority (NI) trial, comparing bilateral single-shot superficial parasternal intercostal plane (SPIP) block with bilateral single-shot erector spinae plane (ESP) block for analgesia after first-time on-pump cardiac surgery via median sternotomy. Blocks are performed pre-induction within a standardised multimodal analgesic pathway. The primary outcome is cumulative intravenous morphine milligram equivalents (IV-MME) from extubation to 24 h post-extubation, with a prespecified NI margin of 3 mg IV-MME. Several methodological and reporting issues require attention.

1."Prolonged postoperative mechanical ventilation (failure to extubate within 8 h after surgery)" is listed under Exclusion criteria, yet this can only be known after surgery and randomisation. Please clarify is this a pre-randomisation screening criterion (in which case it cannot be applied as written), or a post-randomisation handling decision? If the latter, it should be reframed as a prespecified intercurrent-event/analysis-strategy decision (analysed in ITT), not as an eligibility criterion.

2.The primary estimand is described as "the between-group location shift in 24-h IV-MME consumption." For the estimand framework, the protocol should explicitly define the estimand attributes (population, variable, intercurrent-event strategies, summary measure) and how each intercurrent event — death, prolonged ventilation, premature withdrawal, rescue-medication escalation, and block failure is handled.

3.The sample size is derived from a one-sided two-sample t-test (parametric, mean/SD based: margin 3 mg, SD 6.48 mg, 95% power, α = 0.025), whereas the primary analysis uses the Hodges–Lehmann location-shift estimator with a bootstrap/derived CI — a non-parametric, median oriented analysis. The margin and SD themselves are also mean-based historical estimates. Please justify the current sample size confers the intended power for the planned non-parametric NI analysis.

4.The 3 mg IV-MME margin is small (≈0.46 × SD) and represents a clinically very modest quantity of opioid over 24 h. Please state explicitly why a loss of up to 3 mg IV-MME is the largest clinically acceptable difference.

5.Unblinded treating anaesthesiologist may induce performance bias and affect the primary outcome.

6.SPIP is performed as a single injection at one intercostal level (T4–5) per side, whereas a median sternotomy incision typically spans several dermatomes. Please clarify if the intervention achieve the full coverage of the incision.

**Reviewer**	**2**
**Name**	**Tanaka, Kenichi A.**
**Affiliation**	**The University of Oklahoma Health Sciences Center, Anesthesiology**
**Date**	**26-Sep-2026**
**COI**	**NA**

General comments:

This protocol describes a multicentre, assessor-blinded, 1:1 randomised non-inferiority trial comparing a bilateral single-shot superficial parasternal intercostal plane (SPIP) block with a bilateral single-shot erector spinae plane (ESP) block in adults undergoing first-time on-pump cardiac surgery through median sternotomy. The primary outcome is cumulative intravenous morphine milligram equivalent (IV-MME) consumption from extubation to 24 hours, with a non-inferiority margin of 3 mg IV-MME.

The question is timely and clinically useful. Most existing regional analgesia data in cardiac surgery compare a single block against no block, so a direct head-to-head comparison of an anteriorly targeted parasternal technique against the more commonly used ESP block is a

worthwhile contribution. The protocol has real strengths: a multicentre design with center-stratified, web-based concealed allocation; delivery of both techniques by experienced proceduralists (≥50 of each); a standardized anaesthetic and multimodal analgesic pathway; prespecified intention-to-treat and per-protocol analyses, which is correct for a non-inferiority design; a distribution-robust Hodges–Lehmann primary estimator; prospective registration; and a commitment to report regardless of direction. The writing is clear and the structure complete.

My reservations are principally at the level of design logic rather than execution. Below I highlight 3 major points.

Major comments

1. Non-inferiority design and the margin interpretable

Non-inferiority is only meaningful when the active comparator has a reliable, quantifiable effect in the population studied, so that the margin preserves a known fraction of a real benefit (assay sensitivity). Two problems arise. First, the margin is imported from an ESP-block-versus-control meta-analysis and rests on an untested constancy assumption: under the contemporary multimodal regimen and morphine PCA specified here, the absolute ESP effect is plausibly much smaller than in the historical comparison, and this trial contains no no-block or sham arm to verify that either block is doing anything. If both blocks add little on top of multimodal analgesia, "non-inferiority" becomes uninterpretable, and the design cannot distinguish two effective blocks from two ineffective ones. Second, the margin and sample size are built on a mean/normal (two-sample t-test) framework, whereas the primary estimand and analysis are the rank-based Hodges–Lehmann location shift; deriving the standard deviation by dividing a historical oral-morphine SD by a fixed 3:1 conversion compounds this, since opioid conversions are neither linear nor free of uncertainty. Please (i) justify non-inferiority over a superiority framing (or a three-arm design with an internal reference), (ii) defend the constancy assumption explicitly and state the consequence if the contemporary ESP effect is smaller, and (iii) align the margin, power calculation, and primary analysis on a single estimand.

2. Interventions

Both interventions are single-shot bupivacaine 0.25% blocks, with a clinically meaningful sensory effect of roughly 8–14 hours, yet the primary outcome accrues opioid use to 24 hours. For much of that window both blocks have regressed, so a large part of the measured consumption reflects the shared multimodal regimen rather than the block contrast, biasing the comparison toward non-inferiority. More fundamentally, neither a parasternal cutaneous block nor a posterior fascial-plane block reliably covers the deep sternal, mediastinal, pericardial, and chest-drain nociception that contributes substantially to post-sternotomy pain; this shared, uncovered source further compresses any between-block difference in the direction of non-inferiority. Time to extubation is also variable after on-pump surgery, so anchoring the window at extubation makes the block-active fraction of the

window differ across patients. I recommend justifying the 24-hour endpoint against single-shot block duration, adding an earlier block-relevant window (e.g., 0–12 hours) as a co-primary or key secondary outcome, and prespecifying time to extubation as a covariate.

3. Primary outcome

The primary outcome is governed directly by the team titrating PCA and administering rescue opioids, yet the proceduralist is unblinded and no sham injection is placed at the alternate site, so allocation is inferable from injection site and patient positioning. Blinding of outcome assessors and the statistician does not protect an endpoint that the (potentially unblinded) bedside and ICU team control. Opioid consumption is additionally confounded by nausea and vomiting, sedation, and the protocolized analgesic floor, all of which compress variance and can mask true differences. Please specify who titrates PCA and rescue analgesia and whether they are blinded, provide a strictly protocolized and auditable analgesic algorithm to limit discretion, and consider a sham injection at the non-allocated site to preserve blinding of patients and bedside staff.

Minor comments

1. State the planned study start and end dates, as required for protocol papers.

2. Acknowledge that chronic postsurgical pain at 3 months is almost certainly underpowered, and prespecify how it will be analysed and reported.

3. Specify multiplicity control across secondary outcomes, and state whether a claim of analgesic non-inferiority will require concordance between the opioid and pain (NRS/QoR-15) outcomes; non-inferior opioid use with inferior pain scores should not be read as equivalence.

4. Define technical and sensory block-failure criteria and their handling in the ITT and per-protocol populations.

5. Prespecify the adjusted analysis model (center, and any stratification/covariates) to accompany the primary Hodges–Lehmann estimate.

6. Confirm that the per-group evaluable and enrolled figures are internally consistent between the abstract and the sample-size section.

In summary, the clinical question is worth answering and the operational design is sound, but the interpretability of the trial hinges on three linked issues: whether a non-inferiority framework with an externally derived margin is justified without an internal reference, whether the 24-hour opioid window and the shared uncovered pain sources are matched to what single-shot blocks can plausibly influence, and whether the opioid-based primary outcome is adequately protected from performance bias. Addressing these would substantially strengthen the protocol.

**Reviewer**	**3**
**Name**	**Rozec, Bertrand**
**Affiliation**	**University hospital of Nantes, Anesthesiology and critical care**
**Date**	**03-Oct-2026**
**COI**	**NA**

Using this protocol, the authors aim to demonstrate the non-inferiority of two locoregional techniques – the parasternal block and the erector spinae plane block – in reducing post-operative opioid consumption following cardiac surgery

Whilst this protocol raises a question of major clinical interest, unfortunately a number of issues limit the scope of the expected results from the outset

- Why was morphine consumption within 24 hours of extubation chosen as the primary outcome measure? It would seem more relevant to use a more comprehensive score – which the authors, incidentally, propose to test as a secondary outcome – such as the Quality of Recovery-15 questionnaire, and to calculate the required sample size

-Furthermore, for a non-inferiority study, a reference method should be used as the comparator; I am not convinced that the spinal erector block can be regarded as a reference method for cardiac surgery. (Perez-Moreno D et al. J Clin Anesth. 2026 Jun;112:112240).

-It should be noted that the blocks are performed prior to induction of anaesthesia and that their efficacy is assessed: this leads me to two observations:

-Whilst it is commendable to wish to verify the quality and efficacy of regional anaesthesia, in practice – particularly for anterior thoracic blocks – this block will typically be performed after induction. Their position, whilst understandable for an RCT, distances them, in my view, from real-world practice, where many blocks are performed after induction – or where one might debate the merits of performing them before the incision, particularly for preventive analgesia, though that is yet another matter – or at the end of surgery for a more prolonged effect

-Furthermore, the one-off nature of the injection for these regional anaesthetics, when administered early, raises questions about their effectiveness beyond the 6th–8th hour; a catheter would allow for a more prolonged and potentially more effective effect. A third group without regional anaesthesia or receiving a placebo would therefore probably be necessary.

In conclusion: it would undoubtedly be advisable, as a first step, to validate the superiority of the erector spinae block over standard analgesia using a robust criterion (and not merely morphine consumption or a VAS) – something that is not clear in the literature – before

proceeding to a non-inferiority trial with a new technique. Similarly, consideration must be given to ways of prolonging the efficacy of locoregional anaesthesia (catheter, local or systemic adjuvant).

Minor points:

-In their protocol, the authors wish to avoid the use of certain non-opioid analgesics (ketamine, lidocaine); however, they do continue to use dexamethasone – admittedly for the treatment of post-operative nausea and vomiting – which also has analgesic effects that may interfere with their protocol (van Eijk LT et al. BMC Anesthesiol. 2026;26:225).

-The use of halogenated gases to maintain anaesthesia during CPB is not possible in all countries, depending on local practices and regulations in force; it may be advisable to elaborate on this point.

- I am still concerned about the use of remifentanil in cardiac surgery to evaluate postoperative analgesic protocol,. Indeed, remifentanil may be associated with increased morphine consumption and post-operative pain (Zhang B et al. Clin J Pain. 2024;40:187-195; Yang Y et al. BMC Anesthesiol. 2026 ;26:570).

VERSION 1 - AUTHOR RESPONSE

Reviewer 1

Comment 1

Prolonged postoperative mechanical ventilation (failure to extubate within 8 h after surgery)" is listed under Exclusion criteria, yet this can only be known after surgery and randomisation. Please clarify is this a pre-randomisation screening criterion (in which case it cannot be applied as written), or a post-randomisation handling decision? If the latter, it should be reframed as a prespecified intercurrent-event/analysis-strategy decision (analysed in ITT), not as an eligibility criterion.

Response 1

We agree and have removed prolonged postoperative mechanical ventilation from the exclusion criteria. The Primary estimand and Analysis populations sections now state that delayed extubation does not exclude a participant from the randomised-group analysis. For those subsequently extubated, opioid consumption will be measured over the full subsequent 24-hour period, irrespective of the duration of ventilation. Delayed extubation alone will not exclude a participant from the per-protocol population. We have also removed the planned analysis excluding participants extubated after 8 hours. Death before completion of the outcome window is addressed separately.

Comment 2

The primary estimand is described as "the between-group location shift in 24-h IV-MME consumption." For the estimand framework, the protocol should explicitly define the estimand attributes (population, variable, intercurrent-event strategies, summary measure) and how each intercurrent event — death, prolonged ventilation, premature withdrawal, rescue-medication escalation, and block failure is handled.

Response 2

We have added a Primary estimand section defining the target population, treatment conditions, outcome variable, summary measure, and handling of intercurrent events. The summary measure is now the between-group mean difference in cumulative IV-MME, expressed as SPIP minus ESP. Block failure, crossover, and rescue-analgesia escalation are handled using a treatment-policy strategy; rescue opioids contribute to the outcome. Delayed extubation shifts the start of the full assessment window and is not an exclusion. Follow-up continues after intervention discontinuation when consent permits, while loss of permission to collect further data is addressed as missingness.

Death before extubation or completion of the outcome window is distinguished from missing data among survivors. No complete 24-hour value is assigned or imputed after death. The revised manuscript explicitly describes the opioid comparison as conditional on survival through the assessment window and acknowledges post-randomisation selection. All randomised participants remain accounted for in trial reporting, including mortality and safety reporting, but the opioid estimate is not an unconditional treatment effect in every randomised participant. Among survivors, the plan specifies available-case estimation, multiple-imputation sensitivity analysis, and a delta-adjusted tipping-point analysis.

Comment 3

The sample size is derived from a one-sided two-sample t-test (parametric, mean/SD based: margin 3 mg, SD 6.48 mg, 95% power, $\alpha = 0.025$), whereas the primary analysis uses the Hodges–Lehmann location-shift estimator with a bootstrap/derived CI — a non-parametric, median oriented analysis. The margin and SD themselves are also mean-based historical estimates. Please justify the current sample size confers the intended power for the planned non-parametric NI analysis.

Response 3

We agree that the original mean-based calculation did not establish the power of a Hodges–Lehmann primary analysis. We have changed the primary analysis to the between-group mean difference, with a two-sided 95% confidence interval calculated using the Welch method. The upper confidence limit will be compared with the 3 mg IV-MME margin. A bootstrap interval targets the same mean difference; the Hodges–Lehmann estimate is now supportive and will not determine the primary NI conclusion.

Comment 4

The 3 mg IV-MME margin is small (≈0.46 × SD) and represents a clinically very modest quantity of opioid over 24 h. Please state explicitly why a loss of up to 3 mg IV-MME is the largest clinically acceptable difference.

Response 4

The Non-inferiority design and margin section now distinguishes the historical calculation from the investigators' clinical judgement. The 3 mg threshold is the maximum additional mean IV-MME consumption considered acceptable in exchange for the practical advantage of supine SPIP placement. We explain its magnitude using three delivered 1 mg PCA boluses, without treating this illustration as a validated minimal clinically important difference or an acceptable increase for every individual. Preservation of approximately 56% of the conservative historical estimate is explicitly conditional on that estimate being applicable to the current setting. Opioid NI will not be equated with equivalent pain relief or recovery.

Comment 5

Unblinded treating anaesthesiologist may induce performance bias and affect the primary outcome.

Response 5

The Blinding section now separates block delivery from subsequent care. The proceduralist will not manage intraoperative anaesthesia, postoperative ICU care, or outcome assessment. The intraoperative anaesthesiologist and ICU analgesia team will be blinded. Allocation is withheld from routine handover and procedure-specific documentation is kept separate, while safety information remains accessible. Necessary unblinding will be documented. The rescue protocol now specifies 1 mg IV morphine, reassessment after 15 minutes, and safety checks before additional dosing. Participants remain unblinded, and inadvertent disclosure is acknowledged as a limitation.

Comment 6

SPIP is performed as a single injection at one intercostal level (T4–5) per side, whereas a median sternotomy incision typically spans several dermatomes. Please clarify if the intervention achieve the full coverage of the incision.

Response 6

The SPIP description now explains the intention to obtain multisegmental coverage through cranio-caudal spread, citing prior sensory-mapping research reporting T2–T6 involvement. Complete coverage is not assumed. The Methods specify a single injection at the fourth intercostal space on each side for SPIP and at the T5 transverse process on each side for ESP. The cold test identifies a cutaneous response in the assessed region; it does not establish full incision coverage or analgesia for every source of post-sternotomy pain. We retain bilateral single-level injections and do not introduce formal dermatome-by-dermatome mapping.

Reviewer 2

Comment 1

1. Non-inferiority design and the margin interpretable

Non-inferiority is only meaningful when the active comparator has a reliable, quantifiable effect in the population studied, so that the margin preserves a known fraction of a real benefit (assay sensitivity). Two problems arise. First, the margin is imported from an ESP-block-versus-control meta-analysis and rests on an untested constancy assumption: under the contemporary multimodal regimen and morphine PCA specified here, the absolute ESP effect is plausibly much smaller than in the historical comparison, and this trial contains no no-block or sham arm to verify that either block is doing anything. If both blocks add little on top of multimodal analgesia, "non-inferiority" becomes uninterpretable, and the design cannot distinguish two effective blocks from two ineffective ones. Second, the margin and sample size are built on a mean/normal (two-sample t-test) framework, whereas the primary estimand and analysis are the rank-based Hodges–Lehmann location shift; deriving the standard deviation by dividing a historical oral-morphine SD by a fixed 3:1 conversion compounds this, since opioid conversions are neither linear nor free of uncertainty. Please (i) justify non-inferiority over a superiority framing (or a three-arm design with an internal reference), (ii) defend the constancy assumption explicitly and state the consequence if the contemporary ESP effect is smaller, and (iii) align the margin, power calculation, and primary analysis on a single estimand.

Response 1

We agree that the applicability of the historical ESP-versus-control effect is central to the interpretation of this non-inferiority trial. We retain the two-arm design because our clinical question concerns whether SPIP can provide an alternative to ESP without an unacceptable increase in opioid requirements, while allowing block placement in the supine position.

The 3 mg IV-MME margin was derived from the overall meta-analysis by Wang et al. The conservative confidence limit of the historical benefit was 20.57 mg OME, corresponding to 6.86 mg IV-MME under the prespecified 3:1 conversion. Preserving 50% of this estimate yielded 3.43 mg, which was rounded down to 3 mg. The selected margin therefore corresponds to approximately 56% preservation of that historical estimate, conditional on its applicability to the present setting. We additionally examined Wang et al.'s published sensitivity analysis excluding continuous ESP techniques (Supplementary Figure 15). Across four single-shot trials, the mean reduction was 31.62 mg OME (95% CI, 16.39–46.85). Thus, the opioid-sparing finding was not dependent solely on continuous catheter administration. However, using the conservative confidence limit from this analysis and the same conversion, a 3 mg margin would correspond to approximately 45% effect preservation, rather than 50%. We have clarified this distinction and do not claim that the single-shot analysis independently confirms the original preservation percentage. We retain 3 mg as the maximum additional mean 24-hour IV-MME consumption considered clinically acceptable by the investigators in exchange for the practical advantage of supine SPIP placement. This clinical judgement complements the historical calculation; it is not a validated minimal clinically important difference.

We acknowledge that substantial heterogeneity remains in the single-shot analysis (I^2=95%) and that postoperative and post-extubation assessment windows are not interchangeable. These differences prevent assurance that the historical ESP benefit will be reproduced under our multimodal regimen. Without a no-block control, the trial cannot establish either block's absolute benefit. Accordingly, any non-inferiority finding will be interpreted as a comparison of opioid requirements under the specified study conditions and will not, by itself, establish preservation of analgesic benefit over no block or equivalent overall analgesia.

Comment 2.

Interventions

Both interventions are single-shot bupivacaine 0.25% blocks, with a clinically meaningful sensory effect of roughly 8–14 hours, yet the primary outcome accrues opioid use to 24 hours. For much of that window both blocks have regressed, so a large part of the measured consumption reflects the shared multimodal regimen rather than the block contrast, biasing the comparison toward non-inferiority. More fundamentally, neither a parasternal cutaneous block nor a posterior fascial-plane block reliably covers the deep sternal, mediastinal, pericardial, and chest-drain nociception that contributes substantially to post-sternotomy pain; this shared, uncovered source further compresses any between-block difference in the direction of non-inferiority. Time to extubation is also variable after on-pump surgery, so anchoring the window at extubation makes the block-active fraction of the window differ across patients. I recommend justifying the 24-hour endpoint against single-shot block duration, adding an earlier block-relevant window (e.g., 0–12 hours) as a co-primary or key secondary outcome, and prespecifying time to extubation as a covariate.

Response 2

We agree that the duration of single-shot blocks and the interval between block placement and extubation are important to interpretation. We retain cumulative 24-hour post-extubation opioid consumption as the primary outcome because the trial evaluates overall opioid requirements during the first day of awake recovery within a shared multimodal regimen, rather than uninterrupted sensory blockade. Previous trials by Athar et al. and Öğür et al. reported reduced first-day opioid requirements after single-shot ESP blocks, although their regimens and assessment windows are not identical to ours.

In response to the reviewer's recommendation, we have added cumulative opioid consumption during the first 12 hours after extubation as a secondary outcome. This will include PCA-delivered morphine and all additional opioids administered during that period, expressed as IV-MME. The between-group mean difference will be reported with a two-sided 95% confidence interval. No separate non-inferiority margin will be applied, and this analysis will not determine the primary non-inferiority conclusion. The outcome definitions, assessment schedule and analysis plan have been updated accordingly.

We acknowledge that delayed extubation may also shift the 0–12-hour window beyond the period of greatest block effect; this additional outcome therefore does not fully resolve the timing limitation. Time to extubation remains a reported outcome but will not be included in the prespecified adjusted model because it is a post-randomisation variable potentially

influenced by treatment. Neither block can be assumed to cover all deep sternal, mediastinal, pericardial or drain-related pain. Shared multimodal analgesia and regression of block effects may attenuate between-group differences and favour non-inferiority. These limitations will qualify interpretation of the findings.

Comment 3

The primary outcome is governed directly by the team titrating PCA and administering rescue opioids, yet the proceduralist is unblinded and no sham injection is placed at the alternate site, so allocation is inferable from injection site and patient positioning. Blinding of outcome assessors and the statistician does not protect an endpoint that the (potentially unblinded) bedside and ICU team control. Opioid consumption is additionally confounded by nausea and vomiting, sedation, and the protocolized analgesic floor, all of which compress variance and can mask true differences. Please specify who titrates PCA and rescue analgesia and whether they are blinded, provide a strictly protocolized and auditable analgesic algorithm to limit discretion, and consider a sham injection at the non-allocated site to preserve blinding of patients and bedside staff.

Response 3

The revised Blinding section confirms that the clinicians managing intraoperative anaesthesia and postoperative PCA/rescue analgesia will be blinded, although the proceduralist and participants cannot be. The proceduralist takes no part in subsequent care. Rescue morphine is standardised to 1 mg IV for persistent NRS ≥4 despite appropriate PCA use, followed by reassessment at 15 minutes. Repeat doses require review of recent PCA/rescue exposure, blood pressure, respiratory rate, oxygenation and RASS. The manuscript defines withholding and physician-review criteria and records doses, times, assessments and deviations.

We considered a sham injection at the alternate site but elected not to expose participants to an additional needle procedure without direct therapeutic benefit. We acknowledge that separate blinded care teams do not eliminate bias from unblinded participants. Nausea, vomiting and sedation will be recorded, and lower opioid consumption will not be interpreted in isolation as better analgesia. These measures limit discretion but cannot remove every influence of adverse effects or the background regimen on opioid use.

Minor comments

Comment 1.

State the planned study start and end dates, as required for protocol papers.

Response 1.

The Study design section now gives December 2026 to July 2027 for recruitment and December 2027 for completion of final 3-month follow-up. Recruitment has not started.

Comment 2.

Acknowledge that chronic postsurgical pain at 3 months is almost certainly underpowered, and prespecify how it will be analysed and reported.

Response 2.

The manuscript now identifies CPSP as exploratory and states that the trial is not specifically powered for this outcome. Group proportions, risk differences and 95% confidence intervals will be reported; deaths and missing assessments will not be classified as absence of pain. BPI and S-LANSS among participants reporting pain will be summarised descriptively. A non-significant result will not be interpreted as equivalence.

Comment 3.

Specify multiplicity control across secondary outcomes, and state whether a claim of analgesic non-inferiority will require concordance between the opioid and pain (NRS/QoR-15) outcomes; non-inferior opioid use with inferior pain scores should not be read as equivalence.

Response 3.

We have revised the Statistical analysis section to specify Bonferroni adjustment across three prespecified secondary comparisons: TWA NRS pain intensity during coughing over 0–24 hours, cumulative 0–12-hour post-extubation IV-MME consumption, and QoR-15 scores at 24 hours. These comparisons address pain during coughing, earlier opioid requirements and recovery within the first 24 hours after extubation. The family-wise error rate within this family will be controlled at a two-sided level of 0.05. Bonferroni-adjusted p values will be reported alongside effect estimates and nominal 95% confidence intervals. Other outcomes, including resting pain and QoR-15 at hospital discharge, will be interpreted descriptively.

Formal non-inferiority testing is restricted to the primary 24-hour opioid-consumption outcome and requires concordant findings in the intention-to-treat and per-protocol analyses, subject to the prespecified estimand provisions. No non-inferiority margins or joint decision rule have been specified for NRS or QoR-15. Meeting the primary criterion will therefore establish non-inferiority only for opioid consumption under the study conditions. Evidence of worse pain or recovery would preclude interpreting this finding as comparable overall analgesia, even if the corresponding comparison does not meet the multiplicity-adjusted threshold. Non-significant secondary comparisons will not establish equivalence.

Comment 4.

Define technical and sensory block-failure criteria and their handling in the ITT and per-protocol populations.

Response 4.

Technical failure is defined as inability to complete the bilateral assigned injections in the intended planes. Sensory responses are assessed separately on each side. Bilateral absence constitutes sensory failure; unilateral response and an unavailable assessment are identified separately. These definitions are now aligned between the Methods and Table 2. All are retained according to allocation for the randomised-group analysis, subject to the stated estimand provisions. Technical failure is a major deviation for PP analysis. Absence of a sensory response alone does not exclude an otherwise protocol-compliant bilateral procedure from PP, avoiding restriction to physiological responders.

Comment 5.

Prespecify the adjusted analysis model (center, and any stratification/covariates) to accompany the primary Hodges–Lehmann estimate.

Response 5.

The adjusted supportive analysis is now specified as linear regression with treatment and centre as categorical fixed effects; age and BMI as continuous variables; and sex, planned isolated CABG versus other procedure, planned internal mammary harvesting, and baseline chronic pain as categorical covariates. HC3 standard errors will be used. There will be no significance-based variable selection. The analysis targets the same mean difference as the unadjusted primary comparison. The Data collection section now explicitly includes baseline chronic pain status and planned and actual procedure type and internal mammary artery harvesting.

Comment 6.

Confirm that the per-group evaluable and enrolled figures are internally consistent between the abstract and the sample-size section.

Response 6.

The abstract specifies 308 participants, with 154 assigned to each group, consistent with the Sample-size calculation section. That section specifies a requirement of 123 evaluable participants per group before the attrition allowance. The attrition adjustment is 123/0.80 = 153.75, rounded up to 154.

Reviewer 3

Comment 1.

Why was morphine consumption within 24 hours of extubation chosen as the primary outcome measure? It would seem more relevant to use a more comprehensive score – which the authors, incidentally, propose to test as a secondary outcome – such as the Quality of Recovery-15 questionnaire, and to calculate the required sample size

Response 1.

We agree that QoR-15 provides important patient-centred recovery information and retain it as a secondary outcome. Our primary question, however, concerns whether SPIP results in an unacceptable increase in opioid requirements compared with ESP within the same multimodal pathway. Changing the primary outcome to QoR-15 would address broader recovery and require a separately justified NI margin and sample-size calculation. We retain the opioid endpoint while recognising the influences of pain, adverse effects and PCA use. The primary finding will be interpreted alongside pain scores, QoR-15 and opioid-related adverse effects.

Comment 2.

-Furthermore, for a non-inferiority study, a reference method should be used as the comparator; I am not convinced that the spinal erector block can be regarded as a reference method for cardiac surgery. (Perez-Moreno D et al. J Clin Anesth. 2026 Jun;112:112240).

Response 2.

We agree that ESP should not be described as an established gold standard. It is selected as an active comparator on the basis of clinical use and prior randomised evidence in sternotomy. The Perez-Moreno et al. review cited by the reviewer reports modest early analgesic benefit and reduced opioid use after sternotomy, while no clear benefit was seen in minimally invasive cardiac surgery. Our population undergoes median sternotomy. This supports comparator relevance, but not a guarantee of constancy or assay sensitivity in our trial. The Discussion now explicitly states these limitations and the restricted interpretation of an NI result.

Comment 3.

It should be noted that the blocks are performed prior to induction of anaesthesia and that their efficacy is assessed: this leads me to two observations:

-Whilst it is commendable to wish to verify the quality and efficacy of regional anaesthesia, in practice – particularly for anterior thoracic blocks – this block will typically be performed after induction. Their position, whilst understandable for an RCT, distances them, in my view, from real-world practice, where many blocks are performed after induction – or where one might debate the merits of performing them before the incision, particularly for preventive analgesia, though that is yet another matter – or at the end of surgery for a more prolonged effect

Response 3.

Pre-induction placement provides a common timing strategy, permits cold-sensation assessment before general anaesthesia, and completes block placement before systemic heparinisation. The trial does not compare timing strategies or claim superiority over post-induction or end-of-surgery blocks. We acknowledge that earlier administration may shorten the remaining postoperative effect and that the findings are specific to the timing studied.

Comment 4.

Furthermore, the one-off nature of the injection for these regional anaesthetics, when administered early, raises questions about their effectiveness beyond the 6th–8th hour; a catheter would allow for a more prolonged and potentially more effective effect. A third group without regional anaesthesia or receiving a placebo would therefore probably be necessary.

Response 4.

We agree that continuous techniques or additional adjuvants may prolong analgesia. Introducing catheters would change the intervention to include catheter maintenance and infusion management. The present question concerns bilateral single-shot SPIP versus ESP, so these interventions are retained. We do not claim equivalence to continuous techniques or uninterrupted 24-hour sensory blockade. Both study blocks already include adrenaline, and IV dexamethasone is part of the shared perioperative regimen; neither is assumed to guarantee prolonged efficacy.

We retain the two-arm comparison rather than adding a no-block group. This limits the question to relative opioid requirements and prevents demonstration of either block's absolute benefit. We recognise that block regression and shared analgesia can compress differences towards NI. Earlier literature supports potential opioid sparing, but does not eliminate the need for caution about the comparator's effect in the contemporary regimen.

Comment 5.

In conclusion: it would undoubtedly be advisable, as a first step, to validate the superiority of the erector spinae block over standard analgesia using a robust criterion (and not merely morphine consumption or a VAS) – something that is not clear in the literature – before proceeding to a non-inferiority trial with a new technique. Similarly, consideration must be given to ways of prolonging the efficacy of locoregional anaesthesia (catheter, local or systemic adjuvant).

Response 5.

We acknowledge the reviewer's concern that uncertainty about the active comparator's benefit limits the interpretation of a non-inferiority trial. We retain the direct two-arm comparison on the basis of existing randomised evidence of opioid sparing with ESP in sternotomy populations. However, this evidence does not establish its effect under the present regimen. The revised Discussion therefore explicitly acknowledges the unverified constancy assumption and the inability of the trial to establish either block's absolute benefit. Our reasons for retaining single-shot techniques are provided in Response 4.

Minor point 1.

In their protocol, the authors wish to avoid the use of certain non-opioid analgesics (ketamine, lidocaine); however, they do continue to use dexamethasone – admittedly for the treatment of post-operative nausea and vomiting – which also has analgesic effects that may interfere with their protocol (van Eijk LT et al. BMC Anesthesiol. 2026;26:225).

Response 1.

We agree that dexamethasone has analgesic effects in addition to its antiemetic action. It is retained at 8 mg intravenously at induction as a standardised component of PONV prophylaxis in both groups. Although administration is identical between groups, its analgesic contribution may attenuate differences in opioid consumption and potentially favour a finding of non-inferiority. We have explicitly acknowledged this limitation in the Discussion.

Minor point 2.

The use of halogenated gases to maintain anaesthesia during CPB is not possible in all countries, depending on local practices and regulations in force; it may be advisable to elaborate on this point.

Response 2.

We agree that volatile delivery during CPB depends on local regulations, institutional practice and compatible perfusion equipment. The Perioperative management section now explicitly states that volatile anaesthesia will be maintained during CPB in both treatment groups at all participating centres. It also specifies that each centre will confirm compatibility of its delivery equipment and monitoring, and compliance with local requirements, before recruitment. This is a setting-specific feature of our protocol, not a claim that volatile delivery is universally feasible or required. We acknowledge that applicability to centres using intravenous maintenance during CPB may be limited. The 2024 EACTS/EACTAIC/EBCP CPB guideline recognises volatile maintenance as an option rather than a universal requirement.

Minor point 3.

I am still concerned about the use of remifentanil in cardiac surgery to evaluate postoperative analgesic protocol,. Indeed, remifentanil may be associated with increased morphine consumption and post-operative pain (Zhang B et al. Clin J Pain. 2024;40:187-195; Yang Y et al. BMC Anesthesiol. 2026 ;26:570).

Response 3.

We acknowledge the concern. The meta-analysis by Zhang et al. reports greater postoperative morphine consumption with remifentanil in cardiac surgery, so common use in both arms does not make its effects irrelevant. We retain remifentanil within the shared anaesthetic regimen, with titration by an allocation-blinded anaesthesiologist in the prespecified range. Intraoperative opioid administration is already a recorded variable. Actual exposure may differ between participants and groups; uniform dosing ranges do not guarantee equal exposure or eliminate an interaction with the block. The Data collection section includes intraoperative opioid administration and use of analgesic adjuncts.

Our comparison therefore concerns the two blocks within this remifentanil-based pathway and does not establish their effects under a different intraoperative opioid regimen. We do not propose routine adjustment of the primary estimate for actual remifentanil exposure, which is post-randomisation and may lie on the treatment pathway.

VERSION 2 - REVIEW

**Reviewer**	**1**
**Name**	**Lei, Chong**

Affiliation **Fourth Military Medical University, Department of Anesthesiology and Perioprative Medicine, Xijing Hospital**

Date **25-Oct-2026**

COI **NA**

The protocol made some revisions based on the comments. Regarding the trial have not started enrolling patient yet, it is ok with the revisions providing the corresponding revision be approved by local IRB.

Reviewer **3**

Name **Rozec, Bertrand**

Affiliation **University hospital of Nantes, Anesthesiology and critical care**

Date **19-Oct-2026**

COI **NA**

The authors answered my questions and made the expected corrections
