# Supplementary material for: Comparative analgesic efficacy of superficial parasternal intercostal plane block versus erector spinae plane block after cardiac surgery in Türkiye: protocol for a multicentre randomised non-inferiority trial
Source: BMJ Open. 2026 Nov 9;16(11):e127382. doi: 10.1136/bmjopen-2026-127382 (PMC13756325; doi:10.1136/bmjopen-2026-127382)
Supplement: Supplementary file 2 [file bmjopen-16-11-s001.docx]

# Supplementary Table 1. Prespecified outcome definitions

| **Outcome** | **Definition** |
| --- | --- |
| **24-hour opioid consumption** | Total opioid dose administered from extubation to 24 hours after extubation, converted to intravenous morphine milligram equivalents. |
| **12-hour opioid consumption** | Total opioid dose administered from extubation to 12 hours after extubation, converted to intravenous morphine milligram equivalents |
| **Pain intensity** | Time-weighted average NRS pain intensity at rest and during coughing over 0–24 hours after extubation, calculated from assessments at 0, 6, 12, and 24 hours using the trapezoidal method. NRS ranges from 0 (no pain) to 10 (worst pain imaginable). |
| **QoR-15** | Validated 15-item patient-reported quality-of-recovery questionnaire; total score 0–150, with higher scores indicating better recovery. |
| **Rescue analgesia** | Additional intravenous morphine in 1 mg increments for persistent NRS ≥4 despite appropriate PCA use, with reassessment after 15 minutes and prespecified safety checks before repeat dosing. |
| **PONV** | Five-category verbal scale: 0 none, 1 mild nausea, 2 moderate nausea, 3 one vomiting episode, 4 more than one vomiting episode. |
| **Opioid-related adverse effects** | Pruritus, sedation and respiratory depression occurring during the first 24 hours after extubation, according to prespecified clinical definitions. |
| **Time to extubation** | Time from ICU admission to successful removal of the endotracheal tube. |
| **ICU length of stay** | Time from ICU admission to ICU discharge, in hours. |
| **Hospital length of stay** | Time from date of surgery to hospital discharge, in days. |
| **Technical block failure** | Inability to complete the assigned bilateral injections in the intended fascial planes. |
| **Sensory block failure** | Bilateral absence of a sensory response to cold testing in the assessed target regions 20 minutes after block completion. Unilateral sensory responses and unavailable assessments will be recorded separately. |
| **Block-related complication** | Pneumothorax, vascular puncture or injury, bleeding or haematoma, infection, local anaesthetic systemic toxicity or neurological deficit attributable to the block. |
| **Chronic postsurgical pain** | Pain developing or increasing after surgery, persisting for at least 3 months, localised to the surgical or referred region and not better explained by another cause. |

**Abbreviations:** ICU, intensive care unit; NRS, Numerical Rating Scale; PCA, patient-controlled analgesia; PONV, postoperative nausea and vomiting; QoR-15, 15-item Quality of Recovery questionnaire.
